# Shaping Perpendicular Magnetic Anisotropy of Co_2_MnGa Heusler Alloy Using Ion Irradiation for Magnetic Sensor Applications

**DOI:** 10.3390/s23094564

**Published:** 2023-05-08

**Authors:** Anmol Mahendra, Peter P. Murmu, Susant Kumar Acharya, Atif Islam, Holger Fiedler, Prasanth Gupta, Simon Granville, John Kennedy

**Affiliations:** 1 Robinson Research Institute, Victoria University of Wellington, Wellington 6140, New Zealand; 2 National Isotope Centre, GNS Science, Lower Hutt 5010, New Zealand; 3 The MacDiarmid Institute for Advanced Materials and Nanotechnology, Wellington 6140, New Zealand

**Keywords:** magnetic sensor, magnetic tunnel junction, perpendicular magnetic anisotropy, Heusler alloy, Co_2_MnGa, effective anisotropy energy, ion irradiation, displacement per atom, interface intermixing

## Abstract

Magnetic sensors are key elements in many industrial, security, military, and biomedical applications. Heusler alloys are promising materials for magnetic sensor applications due to their high spin polarization and tunable magnetic properties. The dynamic field range of magnetic sensors is strongly related to the perpendicular magnetic anisotropy (PMA). By tuning the PMA, it is possible to modify the sensing direction, sensitivity and even the accuracy of the magnetic sensors. Here, we report the tuning of PMA in a Co_2_MnGa Heusler alloy film via argon (Ar) ion irradiation. MgO/Co_2_MnGa/Pd films with an initial PMA were irradiated with 30 keV ^40^Ar^+^ ions with fluences (ions·cm^−2^) between 1 × 10^13^ and 1 × 10^15^ Ar·cm^−2^, which corresponds to displacement per atom values between 0.17 and 17, estimated from Monte-Carlo-based simulations. The magneto optical and magnetization results showed that the effective anisotropy energy (*K_eff_*) decreased from ~153 kJ·m^−3^ for the un-irradiated film to ~14 kJ·m^−3^ for the 1 × 10^14^ Ar·cm^−2^ irradiated film. The reduced *K_eff_* and PMA are attributed to ion-irradiation-induced interface intermixing that decreased the interfacial anisotropy. These results demonstrate that ion irradiation is a promising technique for shaping the PMA of Co_2_MnGa Heusler alloy for magnetic sensor applications.

## 1. Introduction

Magnetic sensors are integral to many fields, including industrial, security, military, space and biomedical applications [1,2]. These sensors are based on various principles, such as search coil, anisotropic magnetoresistance, giant magnetoresistance, magnetic tunnel junctions, fluxgate, Hall effect and superconducting quantum interference device systems [3]. Among these, magnetic tunnel junction (MTJ)-based magnetic sensors exploit tunnelling magnetoresistance (TMR), which has revolutionized various fields, from data storage in computer hard disks to medical applications such as biosensors [4,5,6,7,8]. TMR relies on the magnetic properties of the material, such as coercivity and magnetic anisotropy, to detect and measure magnetic fields. The magnetic anisotropy determines the sensitivity, accuracy and the sensing direction of the sensor [9,10]. It is particularly beneficial for various sensing applications for a material to have perpendicular magnetic anisotropy (PMA), such as high-density storage devices and read heads [11], non-destructive testing (NDT)-based magnetic flux leakage sensors [12], high field spin-valve sensors [13], spin-transfer-based devices with ultrafast switching [14], spin-torque-based memristors for neuromorphic computing [15] and so on [16,17,18,19,20]. PMA is particularly beneficial in detecting changes in magnetic flux perpendicular to the sensor plane, making it advantageous for NDT-based magnetic sensors, magnetic read head sensors for hard drives, etc. PMA allows the device to detect fields in a direction perpendicular to its plane. Therefore, it is crucial to develop techniques to effectively shape the PMA of magnetic materials in various magnetic sensors and related applications. By tuning the PMA, it is possible to enhance the performance of magnetic sensors, making them suitable for a wide range of applications. For example, a reduced PMA is needed to efficiently switch magnetization using voltage-induced strain, whereas a relatively high PMA is required in spin-transfer-based devices [21,22]. Furthermore, it is also useful to have spatially varying anisotropies in a magnetic stack for magnetic storage devices [23]. 

Materials showing PMA often constitute at least one of the four naturally occurring ferromagnetic elements—Co, Fe, Ni and Gd. Among them Co-based bi-layer or multilayers have been investigated for PMA-related applications owing to the relatively easier tuning of magnetization and anisotropy when fabricated with noble metals [24]. The common examples include Co/Pd, Co/Pt, Co/Ni, Co/Au and Co/Ag multilayers [25,26,27,28]. Many binary (e.g., CoFe, CoPt, FePd, FePd and Mn_3_Ga) and ternary (e.g., CoFeB, TbCoFe and GdFeCo) compounds are used for the fabrication of MTJs using Al_2_O_3_ and MgO barrier layers and various seed layers, such as Ta, Pd, Pt or Ru [29,30]. PMA can be achieved in various ferromagnetic materials in different ways, such as using nanostructured alumina membranes, antidots and nanoholes [31,32,33,34,35,36,37]. Crystalline MgO is commonly used as a barrier layer in MTJs because it yields high TMR values due to coherent tunneling when the (001) phase is formed. Recently, Heusler alloys have attracted strong attention as promising candidates for magnetic sensors [38] due to their unique properties, including large magnetic moments, their half-metallic nature and perpendicular magnetic anisotropy thin films [39], as well as high spin polarization [40,41,42,43]. Full-Heusler alloys have an X_2_Y_1_Z_1_ composition [44], where X and Y are transition metals, whereas Z is a p-block element. The common Heusler alloy examples include Ru_2_MnZ (where Z is Sn, Sb, Ge, Si) [45], Ag_2_YB (where Y is Nd, Sm, Gd) [46], Ni_2_MnY (where Y is In, Sn, Sb) [47], Ni_2_MnZ (where Z is B, Al, Ga, In) [48], Ni_2_FeZ (where Z is Al, Ga) [48], Fe_2_TiZ (where Z is Al, Si, Sn) [49], Fe_2_MnZ (where Z is Al, Si) [49], Cu_2_MnZ (where Z is Al, In, Sn, Ge) [50], Au_2_MnAl [50], Co_2_MnSi [51], Co_2_FeSi [52], Co_2_MnGa [17] and Co_2_FeAl [53]. Among these, the Co_2_MnGa Heusler alloy has been studied for various applications, such as magnetic sensors [43], waste heat conversion devices [42], optical applications [54] and spintronics [55,56]. However, most studies on Co_2_MnGa are on relatively thick samples or bulk ones, where PMA is either absent or is too low for practical applications [57]. It has been reported that a Co_2_MnGa film below a thickness of 3.5 nm has PMA at a relatively low saturation magnetic field (~50 mT), and a relatively high out-of-plane uniaxial anisotropy energy density of 1.3 erg·cm^−3^ was discovered for a 2.8 nm thin film [55,58,59]. 

Various strategies have been explored to tailor the anisotropy of Heusler alloys, such as strain-induced anisotropy change [60], buffer layer effects [61,62], doping-induced disorder effects [63] and irradiation [8,64,65,66]. Modification of the magnetic properties of various materials via irradiation has been reported for several types of irradiations, such as plasma of various gasses [67,68], ionizing radiation [69,70], high-energy protons and cosmic rays, X-rays [71], neutrons [72] and even several low and swift heavy ion irradiations [73,74]. Among these techniques, ion irradiation is a well-established technique for shaping the magnetic properties of materials due to its high precision and control over the irradiation parameters, such as the ion species, ion energy and ion beam current density [8,64,65,66,75,76]. Ion beam modification is a powerful technique that can modify the magnetic properties of materials and induce a variety of precise structural changes. For example, it can increase the saturated magnetic moments [64], modify the magnetic anisotropy [77], create exotic magnetic nanostructures [78], alter the exchange bias [79,80], tune magnetic transition temperatures [81], reduce the crystal ordering temperature [82,83] and induce or enhance magnetoresistance in a wide range of materials and structures [76,84]. Ion irradiation alters the material’s properties by introducing point defects into the lattice, which also leads to changes in its magnetic properties [85,86]. By selecting the appropriate ion species, energy, current density and number of ions, these defects can be precisely controlled [8,64,65,66]. The selection of ion species is often a critical factor that determines the irradiation effects. Light gas ions such as H [87,88] and N [88]; inert gasses such as He [89], Ne [90] and Ar [91]; and even heavy elements such as Fe [92], Cr [93] and Au [94] have been used for irradiation, each having varied effects on the material. 

Displacement per atom (DPA) has been increasingly used to evaluate ion-irradiation or implantation-induced damage in materials, where the DPA denotes the average number of times that an atom from the substrate lattice is displaced during ion irradiation and accounts for all the parameters to provide a single value that represents the total damage endured by the sample. Particularly in magnetic materials, where the structural changes by irradiation-induced damage result in the modification of magnetic properties, DPA is a valuable parameter and has been employed by various researchers in the field [73,95,96,97]. Park et al. [98] reported that 20 keV proton (p^+^) and Cr^+^ ion irradiation of CoFeB/MgO/CoFeB-based MTJs caused different displacement damages. Proton irradiation up to 1 × 10^18^ p^+^·m^−2^ caused negligible displacement damages on MTJ layers, whereas Cr^+^-irradiation-induced displacement damages reduced the magnetization and magnetoresistance. Xiao et al. [73] focused on the heavy ion irradiation of CoFeB/MgO/CoFeB layers using 3 MeV Ta^2+^ ions. They reported that the DPA of up to 3.5 × 10^−4^ for 1 × 10^11^ ions·cm^−2^ was below the threshold limit for any significant displacement damages, above which the displacement damages caused structural damages at the CoFeB/MgO interface. Fassbender et al. provided detailed reviews of the effects of light ion irradiation on simple magnetic structures and the patterning of magnetic structures in 2003 [66] and 2008 [23], respectively. Recently, our group also reviewed the effects of ion irradiation on magnetic thin films and magnetic tunnel junctions for magnetic sensors [8] and demonstrated that ion irradiation can effectively modify the properties of ferromagnetic and antiferromagnetic thin films, multilayer stacks and magnetic tunnel junctions. We also summarized the effects of ion irradiation on various magnetic properties and discussed the major causes of these changes. However, to the best of our knowledge, and despite its versatility, ion irradiation has not been used to tune the PMA on Co_2_MnGa Heusler films, which presents an exciting opportunity for further research in this area. 

In this study, we aimed to investigate the effect of Ar ion irradiation on the anisotropy of Co_2_MnGa films for magnetic sensor applications. To achieve this, we fabricated thin film stacks of MgO (2 nm)/Co_2_MnGa (3 nm)/Pd (2.5 nm) on thermally oxidized silicon substrates and irradiated them with 30 keV argon ions in a fluence range of 10^13^ Ar·cm^−2^ to 10^15^ Ar·cm^−2^. We analyzed the magnetic properties of these films before and after irradiation using the magneto optical Kerr effect (MOKE) and a superconducting quantum interference device (SQUID) magnetometer. Furthermore, we simulated the ion irradiation using Monte-Carlo-based simulation tools to understand the role of interfaces. Our findings demonstrate that Ar-irradiation-induced damage, estimated from the DPA, leads to significant interface mixing, resulting in the reduction of the PMA in the thin films. Our results suggest that the DPA provides a very good estimate of interface intermixing in tailoring the PMA in Heusler-alloy-based MTJs. Furthermore, by elucidating the underlying mechanisms that govern the effects of ion irradiation on the interfacial properties of these films, our study provides valuable insights for the development of improved magnetic sensors and related applications.

## 2. Materials and Methods

**Figure 1:**
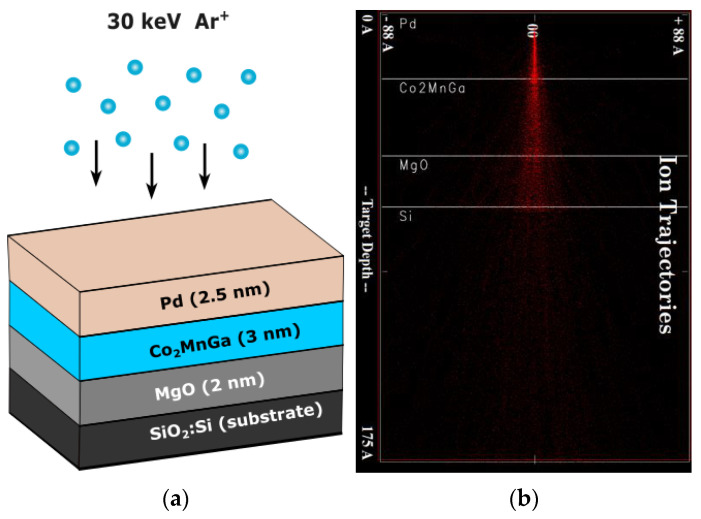
(**a**) The 30 keV ^40^Ar^+^ irradiation of Co_2_MnGa, and (**b**) the SRIM calculation of 30 keV ^40^Ar^+^ into Si/MgO/Co_2_MnGa/Pd.

**Sample preparation:** A Kurt J. Lesker CMS-18 magnetron sputtering system (Jefferson Hills, PA, USA), at the Robinson Research Institute, New Zealand, was used to deposit the thin films. The multilayer stacks were prepared on thermally oxidized silicon substrates measuring 10 mm × 10 mm in the sequence of MgO (2 nm)/Co_2_MnGa (3 nm)/Pd (2.5 nm). The nominal thickness of each layer is indicated in parentheses, and the tri-layer structure is depicted in Figure 1a. The substrate was sourced from the WaferPro LLC (West Palm Beach, FL, USA), MgO from Kurt J. Lesker (Jefferson Hills, PA, USA) and Co_2_MnGa and Pd from AJA International (Country Way, North Scituate, MA, USA). To prevent oxygen interpenetration into Co_2_MnGa, which can reduce the PMA, Co_2_MnGa was deposited above MgO and then capped with Pd [99]. The layers were deposited in a high vacuum chamber with a base pressure of ~1 × 10^−8^ Torr, at an ambient temperature. Subsequently, the stack underwent in situ annealing at 573 K for an hour. RF sputtering was used to deposit MgO at a growth rate of 0.05 Å/s, while Co_2_MnGa and Pd were DC-sputtered at rates of 0.69 Å/s and 4.0 Å/s, respectively, in the absence of an external magnetic field. Growth rates were calculated by measuring the thickness of a thick film (>50 nm) using a Dektak profilometer (Bruker, Billerica, MA, USA) and Rutherford backscattering spectrometry. XRD spectra were not informative for films due to extremely low thicknesses. To verify the composition of the target, we conducted energy-dispersive X-ray (EDX) analysis in a scanning electron microscope (SEM), which confirmed it to be Co_2_MnGa [58]. Further details can be found in the previous report by Ludbrook et al., which detailed that Co_2_MnGa films with thicknesses below 3.5 nm show PMA [58].

**Sample irradiation:** The stack was irradiated using the low-energy ion implanter facility at GNS Science, using a Penning gas ion source [100,101]. The stack was irradiated with 30 keV ^40^Ar^+^ ions in a high vacuum (base pressure ~ 1 × 10^−7^ Torr) at normal incidence and room temperature. The irradiation was performed at a current density of 0.6 µA·cm^−2^ for fluences (ions·cm^−2^) ranging between 1 × 10^13^ and 1 × 10^15^ Ar·cm^−2^. A raster scanning beam was used to obtain the uniform irradiation of the stack. A schematic of the stack and its irradiation is depicted in Figure 1a. We chose the energy for the ions to ensure that most of them would penetrate through the thin films and deposit into the substrate, thereby minimizing implantation effects, as demonstrated in Figure 1b.

**Ion irradiation simulation:** Monte-Carlo-based simulations were performed at default parameters using the Stopping and Range of Ions in Matter (SRIM) [102] and Static and Dynamic Transport of Ions in Matter for Sequential and Parallel computer (SDTRIMSP; Version 5.07) [103] codes, to obtain depth profiles and calculate the displacements per atom (DPA) caused by the irradiation. The DPA was determined using the Kinchin–Pease model [104]. SRIM and SDTRIMSP calculations were performed to obtain the change in atomic concentration with irradiation per nanometer depth and the peak atomic concentrations for the constituent elements, i.e., Mg, O, Co, Mn, Ga and Pd. Material densities used for calculations were Pd = 12.02 g·cm^−3^, Co_2_MnGa = 7.79 g·cm^−3^ and MgO = 3.58 g·cm^−3^. SRIM calculations showed that most ions passed through the multilayer stack and were deposited into the substrate, as shown in Figure 1b. The simulations were used to optimize the ion irradiation conditions for tuning the PMA of the Co_2_MnGa thin films and provided a useful guide for the interpretation of the experimental results.

**Sample characterization:** The magnetic properties of the films were measured via the magneto optical Kerr effect (MOKE) using a Vertisis Technology MagVision Kerr System with a green light of wavelength 500 nm and a 20× magnification objective. Polar-MOKE (P-MOKE) measurements were employed to investigate out-of-plane coercivity and anisotropy at room temperature. A magnetic property measurement system (MPMS) superconducting quantum interference device (SQUID) magnetometer from Quantum Design (San Diego, CA, USA) was utilized to perform magnetization measurements at 300 K. MOKE and MPMS data analysis and fit were performed using the Origin Version 2022 software from OriginLab Corporation (Northampton, MA, USA).

## 3. Results

**Figure 2:**
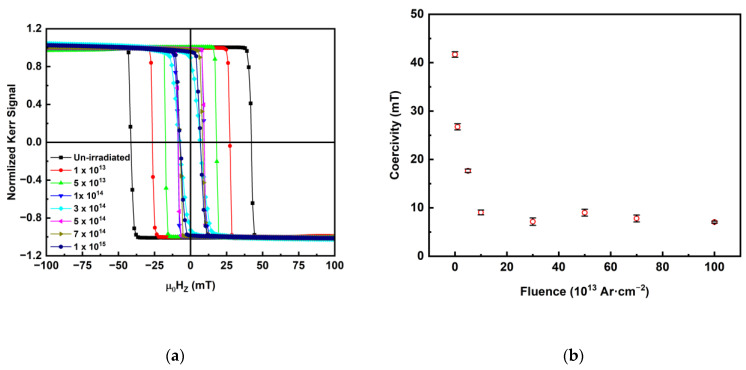
(**a**) P-MOKE hysteresis loops for un-irradiated and Ar-irradiated Co_2_MnGa stacks, and (**b**) coercivity of Ar-irradiated Co_2_MnGa stack.

Figure 2a shows the P-MOKE hysteresis loops for un-irradiated and Ar-irradiated Co_2_MnGa stacks while sweeping an out-of-plane magnetic field within ±130 mT. All the samples showed PMA, which is consistent with our previous studies that showed PMA in Co_2_MnGa thin films below 3.5 nm [58]. The un-irradiated sample showed the presence of very strong PMA, which can be seen by the shape of the hysteresis, and large coercivity of ~42 mT. Upon Ar irradiation, the hysteresis curves became narrow, indicating a decrease in coercivity and PMA. Ar irradiation with fluence 1 × 10^13^ Ar·cm^−2^ decreased the coercive field by ~36% to ~27 mT and further to ~18 mT at fluence of 5 × 10^13^ Ar·cm^−2^. Upon further increasing the fluence, the coercive field was reduced to a certain constant value, with no significant reduction beyond fluence of 3 × 10^14^ Ar·cm^−2^ up to the maximum applied value of 1 × 10^15^ Ar·cm^−2^, as shown in Figure 2b.

**Figure 3:**
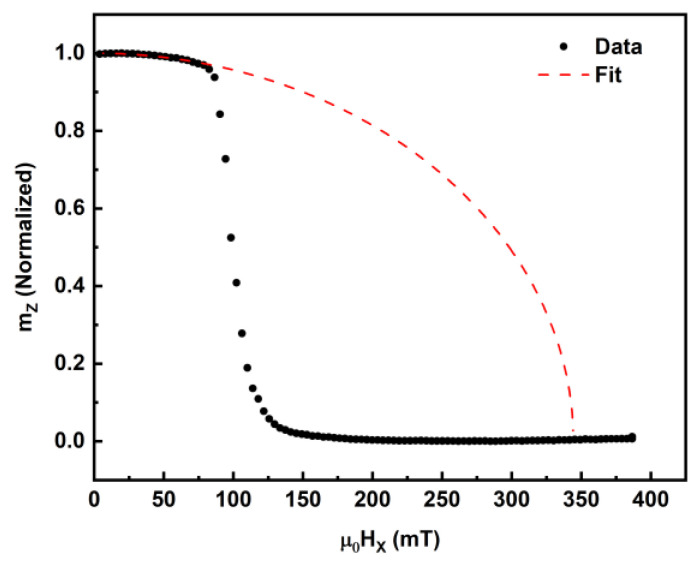
Stoner–Wohlfarth fit of *m_Z_* for 5 × 10^13^ Ar·cm^−2^ irradiated Co_2_MnGa stack.

The out-of-plane uniaxial anisotropy of the stacks was estimated by conducting a magnetic field sweep along the in-plane direction, while measuring the P-MOKE signal. Prior to the in-plane magnetic field sweep, the samples were first saturated along the out-of-plane direction using a magnetic field of 130 mT. Upon reducing the field to zero, the out-of-plane magnetic moment ($m_{Z}$) remained in the out-of-plane direction due to the PMA. The magnetic moment was then rotated from the out-of-plane to the in-plane direction by applying an increasing in-plane magnetic field ($\mu_{0}H_{X}$), as shown in Figure 3.

The effective anisotropy was estimated by applying the Stoner–Wohlfarth model to fit the $m_{Z}$ values and using the saturation magnetization ($M_{S}$) of the stack obtained from SQUID magnetometry [105]. The model assumes that the *y*-axis component of magnetization is zero ($m_{Y}=0$) and the normalized out-of-plane saturation magnetization is 1 ($m_{z}=1$). The *x*-axis component of magnetization can then be calculated using $m_{X}=\sqrt{1-m_{Z}^{2}}$. The Stoner–Wohlfarth equation for a PMA sample can thus be written as

(1) $$m_{Z}=m\sqrt{1-{\left(m\mu_{0}H_{k}/2K_{u}\right)}^{2}}$$

where m is the magnetic moment, *K_u_* is the uniaxial magnetic anisotropy and ($\mu_{0}H_{K}$) is the point where the extrapolated fit crosses $m_{Z}=0$. The magnetic moment rotates coherently in the field regime from 0 to 100 mT, which can be fitted well with the Stoner–Wohlfarth equation, represented by the red dotted line shown in Figure 3. Due the nucleation of magnetic domains at higher external fields, the magnetization deviates from the single-domain Stoner–Wohlfarth behavior. Hence, the effective magnetic anisotropy energy, *K_eff_*, is given by

(2) $$K_{eff}=\frac{\mu_{0}H_{k}M_{S}}{2}$$

**Figure 4:**
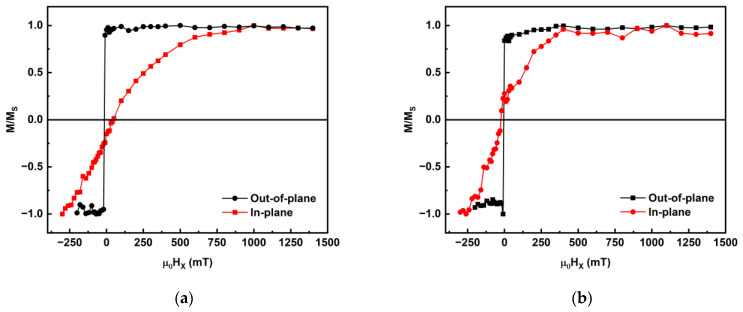

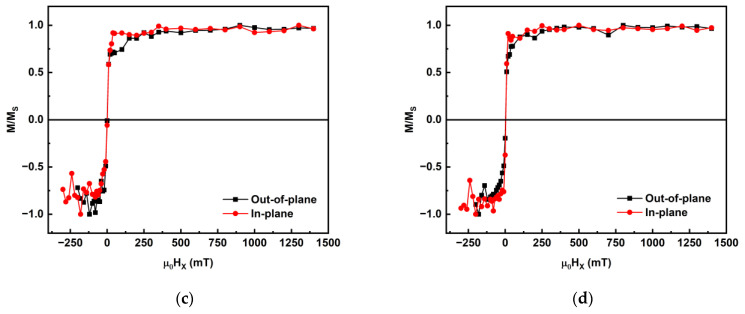
Normalized magnetization vs. field plots for (**a**) un-irradiated, (**b**) 5 × 10^13^ Ar·cm^−2^, (**c**) 3 × 10^14^ Ar·cm^−2^ and (**d**) 1 × 10^15^ Ar·cm^−2^ irradiated Co_2_MnGa stacks.

**Figure 5:**
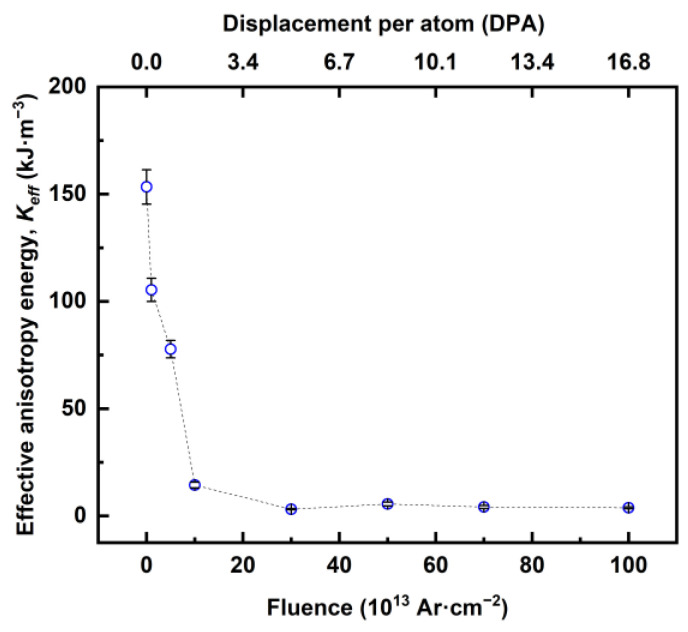
Effective anisotropy energy of Ar-irradiated Co_2_MnGa stacks.

We performed SQUID magnetization measurements along the in-plane and out-of-plane directions of the stacks to determine the saturation magnetization for anisotropy calculation. Figure 4 shows the comparison between in-plane (H_IP_) and out-of-plane (H_OOP_) magnetization normalized to the saturation magnetization, *M_S_*, for the un-irradiated, 5 × 10^13^ Ar·cm^−2^, 3 × 10^14^ Ar·cm^−2^ and 1 × 10^15^ Ar·cm^−2^ irradiated stacks. The curves have had a linear background subtracted from each one to show the component that reaches saturation at low fields < 0.5 T. It is evident that the un-irradiated H_OOP_ curve is along the easy axis and H_IP_ is along the hard axis before irradiation and even for the 5 × 10^13^ Ar·cm^−2^ irradiated stack, which is also consistent with the P-MOKE results, showing strong PMA in this range. It is thus evident that the samples have PMA up to fluence of 3 × 10^14^ Ar·cm^−2^. However, for higher fluences, the H_IP_ and H_OOP_ plots follow a similar trend, indicating a sample with an easy axis both in-plane and out-of-plane. This trend is also validated by the near-zero anisotropy values beyond fluence of 3 × 10^14^ Ar·cm^−2^, as shown in Figure 5.

To simulate and account for the changes in irradiation-induced damage from variations in multiple irradiation parameters, such as ion species, ion energy and fluence, the DPA was calculated. The DPA is a useful metric for comparing damage caused by ion irradiation under different experimental conditions. It provides a normalized value that takes into consideration the ion energy, target properties and irradiation fluence, and allows for comparisons amongst different ion–target combinations [106]. The DPA accounts for all of these parameters to provide a single value that represents the total damage endured by a target surface. Specifically, the DPA is defined as the number of target atoms displaced to a stable interstitial position in the host lattice per incident ion. While it is generally only an approximation, the DPA is widely used in the field of ion-beam materials science. Considering the displacement energy to be E_d_, and the damage energy as E_a_, the DPA is given by [107]

$$\mathrm{DPA}\;=\left\{\begin{array}{c} 0,\;\mathrm{when}\;E_{a}<E_{d}\\ 1\;,\;\mathrm{when}{\;E}_{d}<E_{a}<2E_{d}/0.8\\ 0.8{\;E}_{a}/2E_{d},{\;\mathrm{when}\;E}_{a}>2E_{d}/0.8 \end{array}\right..$$

Of note is the fact that the damage energy E_a_ is the ion energy available to displace atoms by collisions, which is often lower than the actual ion energy due to power lost via collisions during ion irradiation.

The DPA was calculated using the TRIM simulation tool considering the “detailed calculation with full damage cascade” damage [102]. From the total damage plot, the average number of collision events in the Co_2_MnGa layer was taken and the DPA was calculated using the following equation:

(3) $$\mathrm{DPA}=\frac{\mathrm{Fluence}\;\left(\mathrm{ions}\cdot {\mathrm{cm}}^{-2}\right)\times \left(\mathrm{No}.\;\mathrm{of}\;\mathrm{Defects}/Å\;\mathrm{ion}\right)\times 10^{8}}{\mathrm{Atomic}\;\mathrm{Density}\;\left(\mathrm{Atoms}\cdot {\mathrm{cm}}^{-3}\right)}$$

DPA values for 30 keV Ar irradiation for fluence 1 × 10^13^ Ar·cm^−2^ and 1 × 10^15^ Ar·cm^−2^ were calculated to be 0.17 and 17, respectively, where the atomic density of Co_2_MnGa was taken as 2.08 × 10^22^ atoms·cm^−3^. 

The effective magnetic anisotropy energy *K_eff_* was calculated using $\mu_{0}H_{k}$ and *M_S_*, where the anisotropy fields $\mu_{0}H_{k}$ were obtained using MOKE from the SW fit shown in Figure 3 and the *M_S_* for the un-irradiated thin film was obtained from the SQUID measurements in Figure 4. The calculated *K_eff_* values with respect to fluence and DPA are detailed in Figure 5. For the un-irradiated stack, $\mu_{0}H_{k}=526\;\mathrm{mT}$ and $M_{S}=583\;\mathrm{kA}\cdot m^{-1}$, which results in $K_{eff}=153\;\mathrm{kJ}\cdot m^{-3}$. The anisotropy decreased to ≈105 kJ·m^−3^, 77 kJ·m^−3^ and then to 14 kJ·m^−3^ for fluences of 10^13^, 5 × 10^13^ and 1 × 10^14^ Ar·cm^−2^, respectively, and did not change significantly beyond fluence of 3 × 10^14^ Ar·cm^−2^. It is known that ion irradiation can cause an increase in the number of nucleation sites, leading to a reduction in the anisotropy field with fluence [108,109]. This decrease in the anisotropy field consequently results in a reduction in the effective anisotropy, as seen in Figure 5, for the Ar-irradiated stacks. 

These results are consistent with the trend observed in the magnetization measurements obtained from SQUID. Previous research has shown that the PMA in Pd/Co_2_MnGa/MgO occurs due to the interfacial magnetic anisotropy and is present for Co_2_MnGa thicknesses smaller than 3.5 nm [58]. Thus, it is likely that ion irradiation causes intermixing at the interfaces of Co_2_MnGa with Pd and MgO, which increases the interfacial roughness and leads to a reduction in PMA. 

## 4. Discussion

**Figure 6:**
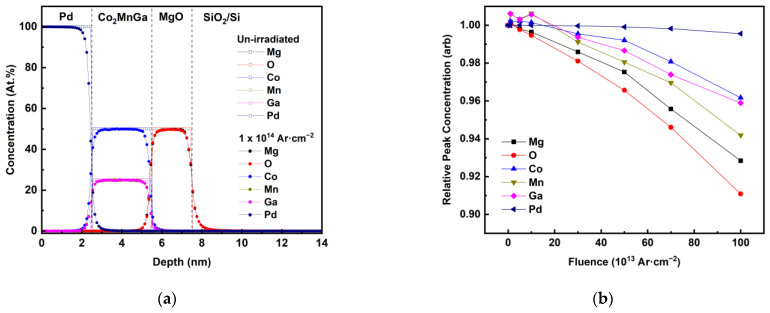
SDTRIMSP calculations of (**a**) depth profiles for an un-irradiated and Ar-irradiated stack of Co_2_MnGa with barrier, buffer and capping layers, and (**b**) peak concentration relative to un-irradiated stack of each element with increasing Ar ion fluence.

To achieve a comprehensive understanding of the impact of ion irradiation on Co_2_MnGa thin films and the interfacial roughening phenomenon, we simulated the process of ion irradiation on Co_2_MnGa using a Monte-Carlo-based simulation code, SDTRIMSP. Using this, we calculated the atomic concentration of each element at a depth resolution of 1 angstrom and were able to obtain insights into the intricate intermixing of various constituent elements at the interfaces. However, it is important to note that the simulations have intrinsic errors related to the various assumptions, pseudo-potentials and stopping power and range values [110]. As expected, we observed an increase in the intermixing of various elements at the interfaces with an increase in fluence, as depicted in Figure 6a. Importantly, the SDTRIMSP calculations further validated the relative intermixing tendencies of different constituent elements, with palladium exhibiting relatively low intermixing levels and magnesium and oxygen displaying the highest levels of intermixing, as anticipated due to their respective atomic masses. This is depicted in Figure 6b as the decrease in the peak atomic concentrations of Mg, O, Co, Mn, Ga and Pd, relative to the un-irradiated stack, with increasing Ar fluence.

In a recent report, Gabor et al. [111] studied CoFe-based Heusler alloys and found that a CoFe-rich interfacial layer promotes strong electronic hybridization between the metal and oxygen orbitals, leading to PMA. Similarly, Sun et al. [62] studied the effects of buffer layers such as Pd, Ru and Cr in the formation of PMA in Co_2_Fe_0.4_Mn_0.6_Si Heusler alloy films. Both groups reported that the interdiffusion of elements and interface roughness during deposition affect the hybridization of CoFe with oxygen and metal orbitals, ultimately leading to PMA. From these studies, it can be inferred that the mechanisms responsible for inducing PMA in CoFe-based non-Heusler thin films with a heavy metal and MgO interface [112] are also applicable to CoFe-based Heusler alloys. While it is well established that Co-, Fe- and CoFe-based non-Heusler alloys exhibit PMA at low film thicknesses, Heusler alloys possess unique properties that make them intriguing for exploring similar applications, potentially paving the way for novel device applications.

We believe that the same mechanisms induce PMA in Co_2_MnGa thin films as suggested for CoFe-based alloys—specifically, Co-3d and Pd-5d hybridization at the Co/Pd interface, and charge transfer between Co and O at the Co/MgO interface, which increases the splitting of out-of-plane hybridized band levels (d_yz_, d_Z_^2^, d_xz_ and p_z_) [99,113,114,115,116]. However, our findings suggest that the observed decrease in PMA observed in Ar-irradiated Co_2_MnGa stacks is primarily due to intermixing at the interface with MgO, as Pd is relatively inert to argon-irradiation-induced intermixing due to its heavy mass, while MgO intermixes relatively easily, as can be seen in Figure 6b. This intermixing induces oxygen penetration into the Co_2_MnGa thin film, which is known to reduce the Co-O hybridization and thus the out-of-plane anisotropy. Our simulations show that the intermixing of various elements at the interfaces increases with fluence, as expected. The different masses of the recoiled ions cause this intermixing, which directly correlates with an increase in interfacial roughness, resulting in a reduction in the PMA. Overall, our results shed light on the underlying mechanisms that govern PMA in Co-based Heusler alloys and provide insights into how to manipulate the PMA for specific applications, such as by controlling the fluence/DPA values of the ion irradiation. With the parameters that we used, our results show that PMA reduces upon Ar irradiation, which is desirable for various applications, such as voltage-tunable sensors, magnetic memory that requires efficient magnetization switching using voltage-induced strain for high-density storage and low power consumption and spintronic devices where a reduced PMA may enhance the stability and controllability of the magnetic domains [21,22,117].

## 5. Conclusions

In summary, we show that ion irradiation is a promising tool to manipulate the magnetic properties of Heusler-alloy-based magnetic sensors. The results indicate that 30 keV argon irradiation in the fluence regime of 10^13^–10^15^ Ar·cm^−2^ can effectively tune the anisotropy of the Co_2_MnGa-based thin films. Monte-Carlo-based simulations estimated the displacement per atom values between 0.17 and 17 for fluences 1 × 10^13^ and 1 × 10^15^ Ar·cm^−2^, respectively. The effective anisotropy energy decreased from *K_eff_* ~ 153 kJ·m^−3^ for the un-irradiated stack to *K_eff_* ~ 14 kJ·m^−3^ for the 1 × 10^14^ Ar·cm^−2^ irradiated stack. The simulations confirmed intermixing at the interfaces due to ion irradiation. We found that the intermixing of Co_2_MnGa with the MgO layer was primarily responsible for the reduction in the PMA, due to the irradiation-induced penetration of oxygen atoms into the Co_2_MnGa thin film. This study highlights the potential of ion irradiation as a localized modification tool to tailor the magnetic properties of Heusler alloys, which can be useful in the development of improved magnetic sensors for various applications.

## Data Availability

Data available on reasonable request.
